# 

**Published:** 1895-08-03

**Authors:** 


**Notices of Births, Deaths, and Marriages** are inserted in this column at a charge of 2s. 6d. for an announcement not exceeding four lines.

**Notice to Advertisers and Subscribers.**

All Advertisements, Orders for Copies of The Hospital and Business Communications should be addressed to **The Manager, NOT TO THE EDITOR,** The Hospital, 428, Strand, London, W.C.

The Cost of Subscription, post free, is as follows:—Per week, 2½d.; per quarter, 2s. 8d.; per half-year, 5s. 3d.; per annum, 10s. 6d. Foreign:—Per Annum, 15s. 2d.; payable, in all cases, in advance.

### NOTICE TO CORRESPONDENTS.

All MS., Letters, Books for Review, and other matters intended for the Editor should be addressed The Editor, The Lodge, Porchester Square, London, W.

The Editor cannot undertake to return rejected MS., even when accompanied by stamped directed envelope.

**"Burdett's Hospital Annual," 1895,**
Now Ready.

Sixth year of publication. 950 pp. Scarlet cloth, gilt lettered. Price 5s. A most complete guide to British, Colonial, Indian, and American Hospitals, Dispensaries, Nursing and Convalescent Institutions, and Asylums. A few copies of the "Annual" for 1894 may still be obtained on application to the Publishers, The Scientific Press (Limited), 428, Strand, London, W.C.

**NOTICE.—Vol. XVII. of "The Hospital" (October, 1894, to April, 1895), in handsome cloth binding, is on sale at the Office of this Journal. Price 6s. Cases for binding the Numbers contained in this Volume, 1s. 6d. Index to Vol. XVII., 2½d., post free.**

**The Monthly Part for August now ready, price 6d.; by post, 8d.**

### The Student of Medicine.

### APPOINTMENTS.

S. J. Aarons, M.B., C.M., appointed Resident Surgeon to the Edinburgh Royal Maternity and Simpson Hospital. A. E. Brindley, M.D Lond., M.B., Ch.B., S.Sc.Vict., appointed Resident Medical Officer to the Manchester Royal Infirmary. J. H. Croom, M.D., F.R.C.P., F.R.C.S.Edin., appointed Physician to the Edinburgh Royal Maternity and Simpson Hospital. W. Doughty, L.R.C.S., L.R.C.P., appointed Medical Officer of Health for the Chillington District of the Kingsbridge Union. A. S. Edwards, M.B., C.M.Edin., appointed Junior Resident House Physician to Smedley's Hydropathic Establishment, and Physician to Smedley Memorial Hospital, Matlock. A. Gooding, L.R.C.P., M.R.C.S., appointed House Surgeon to the London Hospital. H. Horrocks, M.D.Lond., appointed Resident Medical Officer to the Hospital for Consumption and Diseases of the Chest, Brompton, S.W. A. Keith, M.D.Aberd., F.R.C.S.Eng., appointed Senior Demonstrator of Anatomy at the London Hospital Medical School. Dr. A. R. Mackintosh, appointed House Surgeon to the Northern Infirmary, Inverness. F. P. MacLellan, M.B., C.M.Aberd., appointed Medical Officer for the Parish of Assynt and the Quoad Sacra Parish of Stoer, N.B. C. Muirhead, M.D., F.R.C.P.Edin., appointed Consulting Physician to the Chalmers Hospital, Edinburgh. A. J. Nevett, L.R.C.P., L.R.C.S.Edin., appointed Junior Assistant House Surgeon to the Stockport Infirmary. J. L. Russell, M.B., C.M. Edin., appointed Medical Officer for the Cornholme District of the Todmorden Union. J. E. Stuart, L R.C.S., L.R.C.P., appointed Medical Officer of Health for the Borough of Batley. W. M. Taylor, M.B., C.M., appointed Resident Surgeon to the Edinburgh Royal Maternity and Simpson Hospital.

### VACANCIES.

The following vacancies are announced this week, the amount of salary in each case being given after the name of the institution:—

### Resident Medical Officers.

Chichester Infirmary.—House Surgeon. Salary £80 per annum, with board, lodging, and washing. Applications to the Secretary by August 12th.

Durham County Asylum.—Pathologist and Junior Medical Officer. Salary £100 per annum, with board and lodging. Applications to the Superintendent, Durham County Asylum, Winterton, Ferryhill, by August 10th.

Gravesend Hospital.—House Surgeon; doubly qualified. Salary £80 per annum, with board and residence. Applications to Frederick Mitchell, Honorary Secretary, by August 8th.

Lincoln County Hospital.—Assistant House Surgeon. Appointment for six months, but eligible for re-election. An honorarium of £10 for each period of six months, subject to the approval of the Weekly Board, with board, residence, and washing. Applications to the Secretary by August 10th.

### Non-Resident Medical Officers.

Dr. Steevens's Hospital, Dublin.—Ophthalmic Surgeon. Applications to the Secretary before August 7th.

Royal Ear Hospital, Frith Street, Soho Square, W.—Assistant Surgeon. Applications to Donald Murray, Honorary Secretary, by August 10th.

### UNIVERSITIES AND COLLEGES.

### University of Edinburgh.

Examinations.—The Second Professional Examination for Degrees in Medicine began on July 17th, when the written papers on Anatomy and Physiology were set. On July 18th Materia Medica and Pathology, and later in the day the *vivâ voce* examinations were taken up. The First Professional Examination began on July 20th with Practical Chemistry, followed on July 22nd by Chemistry and Botany, on July 23rd by Zoology and Physics, on July 24th by the *vivâ voce* examinations.

University Court.—This Court met on July 15th. A letter was read from the Secretary of the Scottish Universities Commission, informing the Court that the Finance Act, 1895, including the provision for the repeal of the stamp duty on the M.D. Degree, is now law. A letter was read from Major Sprot, thanking the Court for the letter of sympathy which they had sent him on the occasion of the death of his uncle, Dr. Hugh Cleghorn. The Court appointed the following gentlemen to be University Lecturers for the Academical Year 1895-96 in the subjects specified: Mr. C. M. Douglas, B.Sc., in Psychology; Mr. William Peddie, D.Sc., Advanced Experimental Physics; Mr. John Beard, D.Sc., Embryology; Mr. Leonard Dobbin, Ph.D., in Chemical Theory; Dr. Joseph Tillie, in Experimental Pharmacology; Dr. Robert Muir, in Pathological Bacteriology; Dr. David Hepburn, in Regional Anatomy; Dr. A. P. Aitken, in Agricultural Chemistry; and Dr. W. G. Smith, in Plant Physiology. On the recommendation of the Senatus the Court recognised Dr. D. Campbell Black, Anderson's College Medical School, Glasgow, as a teacher whose course of instruction in Physiology qualifies for graduation in Medicine. On the recommendation of the Senatus the Court recognised Dundee University College as an institution the courses of instruction in which qualify for graduation in Science.

### University of Glasgow.

The following have passed the Final Professional Examination for the degrees of Bachelor of Medicine (M.B.) and Master in Surgery (C.M.):—

A. Candidates who took Pathology in Final Professional Examination.—J. C. Abbott, T. Armstrong, F. J. Barker, M.A., T. Baxter, A. Blair, H. A. Böedeker, T. Campbell, V. E. Chang. H. Davies, W. Donaldson, R. J. Edwards, S. English, J. Findlay, R. M. Fraser, M.A., W. Graham, W. Hay, B.D., J. A. Hope, A. B. Hughes, G. Jubb, W. J. Kerr, W. D. Miller, D. M'Donald, W. J. Mackinnon, M. N. MacLay, F. MacRae, E. J. Primrose, M.A., J. Rankin, J. Sandilands.

B. Candidates who Passed Pathology in Third Professional Examination.—J. Adam, W. Alexander, W. Allan, A. R. Anderson, J. Anderson, J. J. S. Anderson, J. L. Anderson, W. S. Baird, R. B. Barr, T. Bell, H. Broom, W. Burns, S. Capie, W. Clow, M.A., B.Sc. F. B. Cormick, S. D. Cowan, M.A., J. Cumming, J. Divine, J. H. Douglas, M. Dunning, J. Ferguson, M.A., W. D. Findlay, A. A. Finkelstein, J. L. Forrest, J. Forster, J. R. Foulds. A. F. Galloway, T. C. Garrett, J. Gillan, M.A., J. D. Graham, J. A. Graham, W. Grove, A. Holt, R. Howie, G. D. Hunter, J. Jack, J. W. Jackson, D. Kerr, D. C. Kirkhope, J Kirkwood, W. H. Lang, B.Sc., W. Lawson, D. Lewis, J. D. Louttit, R. K. Miller, J. M. W. Morison, G. Mowat. A. C. Muir, J. A. D. Mulholland, D. Munro, J. M'Cash, D. M'Coll, W. M. M'Cormick, L. MacLachlan, H. M'Laren, W. Macleod, G. M'Pherson, M.A., A R. Oliver, H. A. Pattullo, J. B. Rae, J. N. Robertson, W. Scobie, B.D., J. Smith, T. O. Speirs, J. Sproull, A. Stevenson, H. Stevenson, A. Stewart, A. H. Stewart, J. Stewart, J. Stirling, H. W. Thomson, J. M. Thornley, C. K. Toland, E. A. Walker, M.A., H. A. Watson, M.A., M. Watson, F. Wolverson, A. Young, B.Sc., J. Young, Glasgow; Emmeline Marie Stuart.

### Society of Apothecaries of London.

The following candidates passed in:—

Surgery.—J. L. Challice, London Hospital; H. H. Thomas, Charing Cross Hospital; A. E. Lovett, London Hospital; C. A. E. Coulthard, St. George's Hospital; M. White, St. Thomas's Hospital; H. G Jones, St. Mary's Hospital; H. C. Watt, Liverpool; H. England, Sheffield; T. J. Gaine, Camb. and St. Mary's Hospital; H. C. T. Dalton, Charing Cross Hospital; J. Cryer, Manchester; and C W. Moorshed, Guy's Hospital.

Medicine, Forensic Medicine, and Midwifery.—W. B. Maurice, St. Mary's Hospital; T. W. Gale, St. Bartholomew's Hospital; W. S. Dibbs, Leeds; C. M. Beadnell, Guy's Hospital; Ethel M. Gough, London School of Medicine for Women; and H C. Watt, Liverpool.

Medicine and Forensic Medicine.—C. C. Preston, Manchester; and B. S. Kesteven, St. Bartholomew's Hospital.

Medicine and Midwifery.—H. W. Ramsay, St. Mary's Hospital.

Medicine.—T. E. H. Keogh, St. Mary's Hospital; R. D. Cox, St. Mary's Hospital; and T. H. P. Peers, Charing Cross Hospital.

Forensic Medicine and Midwifery.—A. P. Eldred, St. Mary's Hospital; H. W. Silver, Charing Cross Hospital; and H. England, Sheffield.

Forensic Medicine.—G. S. Taylor, Manchester; H. E. De Vall, Birmingham; and J. M. A. Lamb, London.

Midwifery.—G. A. Child, St. Thomas's Hospital.

To Messrs. Challice, Thomas, Lovitt, Maurice, Gale, Dibbs, Beadnell, Watt, Kesteven, Keogh, Cox, and Peers was granted the diploma of the Society.

